# Determinants and levels of cervical Cancer screening uptake among women of reproductive age in South Africa: evidence from South Africa Demographic and health survey data, 2016

**DOI:** 10.1186/s12889-021-12020-z

**Published:** 2021-11-05

**Authors:** Monica Ewomazino Akokuwebe, Erhabor Sunday Idemudia, Abiel M. Lekulo, Ogone Warona Motlogeloa

**Affiliations:** North-West University Faculty of Humanities, North-West University, Mafikeng, South Africa

**Keywords:** Cervical cancer, Determinants, Levels, Reproductive age, Women, Screening, South Africa

## Abstract

**Background:**

Cervical cancer (CC) is the cancer with the most incidents and the leading cause of cancer mortality among women in South Africa. CC screening is one of the most cost-effective control approaches for the disease burden. This study assessed the determinants and individual-level indicators of cervical cancer screening uptake among women of reproductive age in South Africa.

**Methods:**

We analyzed data from the 2016 South Africa Demographic Health Survey. Our analysis focused on 5903 women (15–49 years). We conducted Chi-square test for bivariate analysis, and multivariate binary logistics regression was used to analyze independent association between individual-level factors and women who have had Pap smear testing. Statistical significance was set at *p* < 0.05.

**Results:**

The mean age at cervical cancer screening uptake among women in South Africa was 40.8 years (SD 18.6, range 15–95 years). A majority of the women (39.3%) were aged 45 years and above and 54.6% of them resides in urban settlements. About 35.4% of women (*n* = 2098) have had a Pap smear test, with 66.5% of them who had a Pap smear test resides in Western Cape province. The proportion of women who had a Pap smear test was significantly higher among those with higher educational attainment (68.7%, *p* = 0.000), in the rich wealth index (50.1%, p = 0.000), and those with health insurance cover (60.3%, p = 0.000). Pap smear testing was found to be more prevalent among women aged 45^+^ years, were in the white population group, had higher education, were divorced, and had health insurance cover. The predominance of Pap smear test was 14% higher among women who are working in the professional/formal sector (AOR; 1.38, 95% CI; 1.14–1.69). The uptake of Pap smear test was also higher among women aged 35–44 years.

**Conclusions:**

The prevalence of cervical cancer uptake is substantially low among women aged 15–24 years in South Africa and shows a degree of between-provinces differences. Therefore, heath educational interventions aimed at increasing the uptake of cervical cancer screening services in South Africa are critically needed.

## Background

Cervical cancer is a serious public health problem and is one of the second leading causes of cancer-related mortality among women worldwide. Its epidemiology and health impacts are not only affecting women, but also their families, communities and social institutions [1]. Although it is also one of the most preventable disease through proper screening, treatment and follow-up, more than half a million women worldwide develop intrusive cervical cancer each year [2]. Middle- and low-income countries bear an unequal share (83%) of the global cervical cancer burden, but only achieve an average successful screening coverage of 19%, compared to 63% in high-income countries [3]. Studies [4–6] have accounted for over 275,000 female deaths and approximately 529,000 new diagnoses each year; besides, cervical cancer disease burden is more prevalent in older women who are post-childbearing [7–12]. In the developing countries, lack of resources limits coverage of cervical screening for women [13–15], and notably, for those from rural communities compared with urban areas, as the rural population is extensively poor and their access to health services is more difficult [16–22]. Overall, developed countries such as the United States of America (USA) and United Kingdom, 40 and 42% of the women diagnosed with cervical cancer respectively, die from it [7, 10, 11, 22, 23], while in Africa and South Asia, the equivalent death rates are nearly twice (78%) as high as the rates experienced in high-income countries [23, 24].

In sub-Saharan Africa, cervical cancer encompasses 20–25% of all cancers among women. The estimate is double that of women globally, as its incidence ranges from 30 to 40 per 100,000 women [24]. In South Africa, cervical cancer is the cancer with the most incidents and the leading cause of cancer mortality among women [25]. Contemporary estimates have shown that every year in South Africa, 5743 women are diagnosed with cervical cancer and 3027 die from the disease. Also, it is the second most frequent cancer among women of reproductive age between 15 and 44 years of age, after breast cancer. Studies [26–28] have shown that young women are at higher risk as they tend to be sexually active and have higher numbers of sexual partners. Several studies [8, 26, 27] have reported that young women are poorly informed about cervical cancer with its associated risk factors, and are unclear about the intent of cervical cancer screening, as well as holding on to negative or inaccurate beliefs or attitudes to Pap testing. Furthermore, one of the known main causative agents in cervical cancer is HPV and there are over 200 recognized serotypes of the HPV virus. Existing studies [29, 30] have documented that about 21.0% of women in the general population of South Africa are estimated to harbor cervical HPV infection in South Africa. These reports have shown that 62.8% of invasive cervical cancers among women in South Africa are attributed to HPVs 16 or 18 [29], which fall in the high-risk HPV serotypes classification, and which are responsible for almost 70% of cervical cancer cases.

Other factors for increasing young women’s susceptibility to cervical dysplasia (dysplasia is defined as medically unusual growth or unusual development or growth of a part of the body such as an organ, bone or cell, including the total absence of such a part) include smoking, oral contraceptive use, and vulnerability of the adolescent cervix to sexually transmitted infections [31–33]. As a consequence of the sexually transmitted nature of HPV, early exposure to sexual intercourse and multiple sex partners are significant risk factors for cervical cancer [5, 34]. Studies have reported that 80–90% of women will have this sexually transmitted infection at some point in their life, although only 3–4% of them will develop cervical cancer [35, 36]. Earlier studies [29, 37] have indicated that the South African Cancer Association (SACA) report in the year 2006 revealed that the age-standardized incidence rate for cervical cancer was 24.71 per 100,000 population. As a result of SACA reports, the South African Department of Health developed the Cervical Cancer Screening Programme, which allows three Pap smears per lifetime, at 10-year intervals, starting at the age of 30. This screening policy programme was designed for target coverage of at least 70% of women nationally [37].

Even with the implementation of the Cervical Cancer Screening Policy Programme in South Africa, the screening coverage is 20%, nationally low for women over the age of 30 years [38]. A population-based study conducted among rural South African women reported that only 18% of the women had ever had a Pap smear test [39]. Another study conducted among female university students found that 42.9% of the participants had heard of cervical cancer, but only 9.8% of the participants had ever had a Pap smear test [40]. Another study conducted in Vhembe District of South Africa, reported that campaigns and motivational talks on cervical cancer as well as cervical cancer screening services are provided for women in all the clinics in the district [41], yet very few women voluntarily present themselves for cervical cancer screening services.

Moreover, women who utilized the cervical cancer screening services do so because they have been referred by other health facilities, or the women are presenting with symptoms. Factors that are associated with poor uptake of cervical cancer screening services are pain, stigmatization, and fear of embarrassment [42–44], time constraint [25, 45], the related costs [25, 42, 45], insolence of health workers [46], lack of awareness about where to get screening [25, 47] and delays in hospital cancer screening [25]. Therefore, cervical cancer becomes a public health burden in countries where there are no cervical cancer screening services, or poor utilization of screening services. Cervical cancer screening services could be utilized better if awareness campaigns are sustained and the services are accessible, affordable and available [48]. Early screening is proven to be cost-effective and a form of control strategy of the disease burden [6, 33]. Thus, early screening of cervical cancer is an important preventative strategy for the disease burden. Improving screening services will not be sufficient to result in increased screening uptake among women, unless we understand and address the multidimensional causes that are likely to impel women’s disposition towards cervical cancer screening. Very little is known about determinants and levels of cervical cancer uptake among women of reproductive age in South Africa. No previous study conducted in South Africa has investigated the determinants and levels of cervical cancer uptake among women of reproductive age (15–49 years) in South Africa. The objectives of this study were to examine the association between determinants and cervical cancer uptake at the individual- and community-factor levels using a logistic statistical model, and to assess the extent of the variation in the uptake levels of cervical cancer screening services in South Africa. The findings of this study will provide insight into the provision of appropriate educational interventions for disease risk reduction and effective cervical cancer screening uptake among women of reproductive age in South Africa.

## Methods

### Study setting

South Africa, officially the Republic of South Africa (RSA), covering 471,445 km^2^, has a population of approximately 59.62 million people, comprising diverse cultures, religions, origins and languages [49]. South African culture is not homogeneous but rather a collection of cultures, with different cultures being predominant in different regions. The country has an upper middle-income economy identified as newly industrialized country [50]. However, poverty and inequality remain widespread, with about a quarter of the population unemployed and living on less than US$1.25 a day. The country has been identified as a middle power in international affairs, and maintains a significant regional influence [50]. However, wide differences exist in the political and administrative economy of the country, with nine provinces: Western Cape, Eastern Cape, Northern Cape, North West, Free State, KwaZulu-Natal, Gauteng, Limpopo and Mpumalanga [51]. Most provinces, except Limpopo, have high levels of socioeconomic development and tourist attractions in terms of infrastructure, industries and services. Limpopo province is a known typical developing area, with export and import of primary products and manufactured goods and services, as well as a large platinum deposit. Yet it is one of the poorest regions of South Africa, with a big gap between poor and rich residents, especially in rural areas [51].

The country operates a three-tier system of government (Legislative, Executive and Judicial) with an independent judiciary operating in a parliamentary system. The provincial governments of the nine provinces of South Africa have their own executive and legislative branches, but not separate judicial systems [49], while the local government consists of municipalities of various types. The largest metropolitan areas are governed by metropolitan municipalities, while the rest of the country is divided into district municipalities, each of which consists of several local municipalities [49]. This synopsis of political economy underlines vital aspects of culture and contextual influences on people’s lifestyles and behaviours.

### Data and sample

This study used data from the most recent 2016 South African Demographic and Health Survey (SADHS) [51], conducted in South Africa, which is the third DHS, and follows the surveys carried out in 1998 and 2003. The SADHS is a nationally representative survey dataset conducted and collected as a collaboration between Statistics South Africa (Stats SA), the South African Medical Research Council (SAMRC) and the National Department of Health (NDoH), with technical support from ICF through the DHS Program of the United States Agency for International Development (USAID). The survey was designed to provide representative estimates for main demographic and health indicators for the country as a whole, for urban and non-urban areas separately, and for each of the nine provinces in South Africa.

A two-stage stratified sampling design was applied that involved randomly selecting the sampling clusters that were created in the first stage, followed by randomly selecting households in the second stage. Implicit stratification and proportional allocation were achieved at each of the lower administrative levels within a given sampling stratum by sorting the sampling frame according to administrative units at different levels in each stratum and using probability proportional to size selection at the first stage of sampling. Questionnaires were pre-tested to ensure that the questions were clear and could be understood by respondents. Our analysis focused on women of reproductive age (15–49 years) in 5903 clusters who were interviewed face-to-face about cervical cancer.

### Measures

#### Outcome variables

The outcome variable for this study is a Pap smear (Pap smear test is one of the types of test used in cervical cancer screening that is carried out on a sample of cells from the cervix to check for abnormalities that may be indicative of cervical cancer) uptake, which is a binary variable whereby the respondents were asked if they have had a Pap smear test. Specifically, respondents were asked “Have you ever been tested or examined for cervical cancer?” (No/Yes). Respondents who answered “Yes” were then asked “Whether they ever had a Pap smear?”

#### Individual-level variables

Individual-level variables at the micro level included were twenty: women’s age (15–24, 25–34, 34–44 and 45^+^ years), population group (Black African, White, Coloured, and Indian/Asian), province (Western Cape, Eastern Cape, Northern Cape, Free State, KwaZulu-Natal, North West, Gauteng, Mpumalanga, and Limpopo), place of residence (urban and rural), educational level (no education, primary, secondary, and higher), occupation (not working, professional/formal, and non-professional/informal), marital status (never married, married, divorced, and widowed), health insurance cover (yes, and no) and own health perception (poor, average, good, and excellent). The household wealth index was a composite score measured by ownership of household items and facilities based on a DHS-generated quintile index and was categorized as poorest, poorer, middle, richer and richest. The quintile index for poorest and poorer was merged as poor wealth index; and richest and richer was also merged as rich wealth index. In this study, the principal investigators re-categorized the merged DHS-generated quintile index as poor, middle and rich wealth index.

#### Community-level variables

Geographical type and provinces were non-aggregate community-level variables. Geographical type was recorded as urban and rural. Provinces were defined as the region where a woman comes from. Basically, South Africa is demarcated into nine provinces: Western Cape, Eastern Cape, Northern Cape, Free State, KwaZulu-Natal, North West, Gauteng, Mpumalanga, and Limpopo, and the living status of their population, and settings may have a relationship with cervical cancer screening uptake. Another group of community-level variables were constructed through an aggregation from individual-level using an average approaches to conceptualize the neighborhood effect on cervical cancer screening uptake by women of reproductive age. The other group of community-level variables were: women’s age, population group, province, place of residence (urban and rural), educational level, occupation, marital status, health insurance cover, own health perception, and wealth index.

### Statistical analyses

Data were weighted to give an explanation for multistage sample design, and analysed using Stata 14 (StataCorp, 2017). Univariate analysis illustrated frequencies and percentages for socio-demographic variables. Cross-tabulations of each independent variable and ever had a Pap smear were applied for inferential analysis. A chi-squared test ascertained whether there was any association between population characteristics and ever had a Pap smear. Multivariate binary logistics regression using variables whose univariate analysis was significant was used to estimate the independent association between respondents who have had Pap smear testing and population characteristics. Unadjusted and adjusted models were constructed in the logistics binary regression, and only individual variables were included to bring out interested findings for this study. Hence, the odds ratios (ORs) for the binary logistic regression with 95% confidence intervals (95% CIs) were reported.

### Ethical considerations

All data were obtained from the 2016 SADHS. Informed consent was obtained from each respondent before the interviews (2016 SADHS). We obtained approval to use the data from the DHS repository (http://dhsprogram.com/data/available-datasets.cfm).

## Results

Table 1 shows the socio-demographic characteristics of women of reproductive age with a total of 5903 who participated in the study. From the table below, 35.5% of women indicated that they have had a Pap smear test, and 64.5% of them reported that they have not had a Pap smear test. The mean age at cervical cancer screening among women in South Africa was 40.8 years (SD 18.6, range 15–95 years). Most women were 45^+^ years (39.0%) followed by 15–24 years (23.6%) who participated in the survey. A majority of the women were from the African/Black population group (84.9%), with the Indian/Asian population group having the least (1.3%). Out of all the women interviewed, 15.9% of the respondents were from KwaZulu-Natal province, followed by 14.1% from Limpopo province. More women were from the urban areas (54.6%) than rural areas (45.5%) (Table 1).

**Table 1:** Distribution of Population by Socio-Demographic Characteristics, South Africa

Socio Demographic Characteristics		Frequency	Percentage
		***N*** = 5903	100
Ever had Pap Smear	Yes	3805	35.5
	No	2098	64.4
Age Group	15–24	1394	23.6
	25–34	1268	21.5
	35–44	937	15.9
	45+	2304	39.0
Population Group	African/Black	5010	84.9
	White	251	4.3
	Coloured	566	9.6
	Indian/Asian	76	1.3
Province	Western Cape	445	7.5
	Eastern Cape	779	13.2
	Northern Cape	495	8.4
	Free State	637	10.8
	KwaZulu-Natal	941	15.9
	North West	561	9.5
	Gauteng	547	9.3
	Mpumalanga	666	11.3
	Limpopo	832	14.1
Geographical Type	Rural	2683	45.5
	Urban	3220	54.6
Educational Attainment	No education	569	9.6
	Primary	1018	17.3
	Secondary	3776	64.0
	Higher	540	9.2
Occupation	Not working	4127	69.9
	Professional/Formal	1020	17.3
	Non-professional/Informal	756	12.8
Marital Status	Never Married	3667	62.1
	Married	1461	24.8
	Divorced	113	1.9
	Widowed	662	11.2
Wealth Index	Poor	2430	41.2
	Middle	1317	22.3
	Rich	2156	36.5
Health Insurance Cover	Yes	796	13.5
	No	5107	86.5
Perceived Health Status	Poor	766	13.0
	Average	1980	33.5
	Good	2384	40.4
	Excellent	773	13.1

Source: SADHS, 2016

The majority of the respondents had secondary education (64.0%) and 17.3% of them have primary education. Among the women interviewed, 69.9% of the women are not working while 17.3% work in the professional/formal sector and 12.8% work in the non-professional/informal sector. Three thousand six hundred and sixty-seven (62.1%) of them have never been married; most (41.2 and 36.5%) were in the poor and rich wealth index categories respectively (Table 1). The majority (86.5%) of the women do not have health insurance cover and 40.4% perceived themselves to have good health status.

Table 2 presents the results of the bivariate analysis of the individual-level characteristics and Pap smear testing. In the bivariate analysis, all individual-level variables (age group, population group, province, geographical type, educational attainment, occupation, marital status, wealth index, health insurance cover and perceived health status) were significantly associated with women’s responses to cervical cancer screening (Pap smear test). Regarding the age group showing the most significant association with Pap smear testing (χ2 = 724.93, *p* = 0.000), a majority of women (52.5%) in age cohorts of 35–44 years have undertaken a Pap smear test compared to those in other age groups.

**Table 2:** Population Characteristics by Pap Smear Testing among Females Aged 15–49 Years in South Africa

Ever had a Pap Smear					
**Population Characteristics**	**No**		**Yes**		**Total** **5903**
	**N**	**%**	**N**	**%**	
**Women’s age group**			**χ2 = 724.9315; *p* = 0.000**		
15–24	1291	92.6	103	7.4	1394
25–34	839	66.2	429	33.8	1268
35–44	445	47.5	492	52.5	937
45+	1230	53.4	1074	46.6	2304
**Population Group**			**χ2 = 306.7635;** ***p*** **= 0.000**		
African/Black	3442	68.7	1568	31.3	5010
White	59	23.5	192	76.5	251
Coloured	273	48.2	293	51.8	566
Indian/Asian	31	40.8	45	59.2	76
**Geographical Type**			**χ2 = 183.8032; *p* = 0.000**		
Urban	1828	56.7	1392	43.2	3220
Rural	1977	73.7	706	26.3	2683
**Educational Attainment**			**χ2 = 164.2445;** ***p* = 0.000**		
No education	434	76.3	135	23.7	569
Primary	665	65.3	353	34.7	1018
Secondary	2483	65.8	1293	34.2	3776
Higher	223	42.3	317	68.7	540
**Occupation**			**χ2 = 238.79335;** ***p* = 0.000**		
Not working	2903	70.3	1224	29.7	4127
Professional/Formal	462	45.3	558	54.7	1020
Non-professional/Informal	440	58.2	316	41.8	756
**Marital Status**			**χ2 = 327.8518; *p* = 0.000**		
Never Married	2649	72.2	1018	27.8	3667
Married	706	48.3	755	51.7	1461
Divorced	32	28.3	81	71.7	113
Widowed	418	63.1	244	36.9	662
**Wealth Index**			**χ2 = 339.9505; *p = 0.000***		
Poor	1829	75.7	591	24.3	2430
Middle	891	67.7	426	32.4	1317
Rich	1075	49.9	1081	50.1	2156
**Health Insurance Cover**			**χ2 = 246.2157;** ***p* = 0.000**		
No	3489	68.3	1618	31.7	5107
Yes	316	39.7	480	60.3	796

Source: SADHS, 2016

A large proportion of white women (76.5%) have undertaken a Pap smear test compared to women in the other population groups, and this shows a significant association between population group and Pap smear uptake (χ2 = 306.76, *p* = 0.000), while educational attainment was found to be significantly associated with Pap smear testing (χ2 = 306.7635; *p* = 0.000), and 43.2% of urban women have had a Pap smear test compared to their rural counterparts, at only 26.31%.

**Fig. 1:**
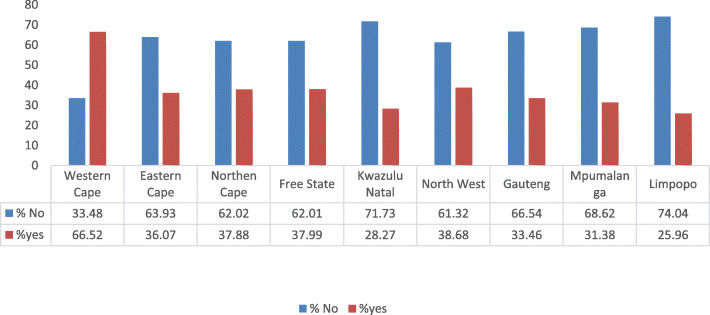
Graph showing proportion of women of reproductive age who have ever had a Pap smear, by province

It can be deduced from the findings that urban women have higher chances of undertaking Pap smear testing, as geographical type was significantly associated with Pap smear testing (χ2 = 183.80; *p* = 0.000) (Table 2). Figure 1 depicts the proportion of women of reproductive age who have had Pap smear by province. Western Cape Province had the highest proportion (66.52%) of women who have had a Pap smear, while Limpopo province had the lowest proportion of women who have had a Pap smear (Fig. 1).

**Fig. 2:**
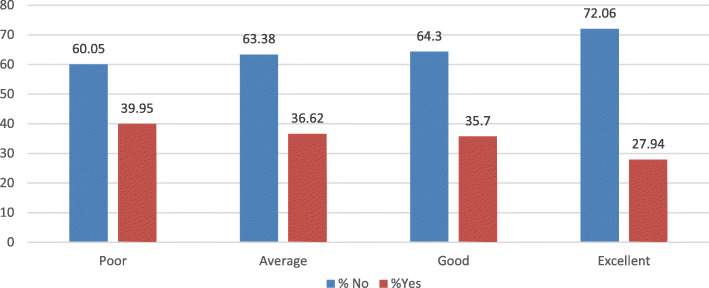
Graph depicting perceived health status of women aged 15–49 years who have ever had a Pap smear

However, a majority of women (39.95%) who perceived their health status to be poor had taken a Pap smear test compared to those with perceived excellent health status (27.94%) (Fig. 2).

Table 3 presented the multivariate analysis illustrating both the unadjusted (U) and adjusted (A) Odds Ratios (OR) of population characteristics which were found to be significant predictors of Pap smear uptake among women of reproductive age. Women aged 35–44 years were found to have 14 and 12% (UOR: 12.14: 95% CI = 9.36–15.72; AOR = 13.62: 95% CI = 10.56–17.55) higher odds of having a Pap smear compared to those aged 15–24 years, and this was found to be statistically significant. Population group has a significant association with Pap smear uptake as the odds of having a Pap smear among white respondents were 2.2 and 2.5 times (UOR; 2.21, 95% CI; 1.54–3.15; AOR; 2.47, 95% CI; 1.75–3.50) higher as compared to African/Black women. Province was significantly associated with lesser odds of having Pap smear uptake: Gauteng (UOR: 0.24, 95% CI: 0.17–0.34; AOR: 0.24, 95% CI = 0.17–0.33); Limpopo (UOR; 0.30, 95% CI; 0.21–0.43; AOR; 0.30, 95% CI; 0.21–0.42); KwaZulu-Natal (UOR; 0.30, 95% CI; 0.22–0.42; AOR; 0.30, 95% CI; 0.21–0.41).

**Table 3:** Logistic Regression Showing Odds Estimates of Pap Smear Testing amongst Women aged 15–49 years by Population Characteristics in South Africa

Population Characteristic	Ever had Pap Smear, ***N*** = 2098							
	Model I: Unadjusted AOR				Model II: Adjusted AOR			
	OR	p > Z	[95% Conf. Interval]		OR	p > Z	[95% Conf. Interval]	
**Age group**								
15–24	**RC**				**RC**			
25–34	5.61	0.000	4.3862	7.17	6.09	0.000	4.78	7.76
35–44	12.14	0.000	9.3698	15.73	13.62	0.000	10.57	17.55
45+	11.95	0.000	9.2343	15.47	13.06	0.000	10.12	16.85
**Population Group**								
African/Black	**RC**				**RC**			
White	2.21	0.000	1.54	3.16	2.48	0.000	1.75	3.51
Coloured	1.05	0.697	0.81	1.37	1.07	0.697	0.82	1.39
Indian/Asian	1.56	0.107	0.91	2.68	1.63	0.107	0.95	2.80
**Province**								
Western Cape	**RC**				**RC**			
Eastern Cape	0.40	0.000	0.29	0.55	0.40	0.000	0.29	0.55
Northern Cape	0.38	0.000	0.28	0.52	0.37	0.000	0.27	0.50
Free State	0.33	0.000	0.24	0.47	0.33	0.000	0.23	0.45
KwaZulu-Natal	0.31	0.000	0.22	0.43	0.30	0.000	0.22	0.42
North West	0.46	0.000	0.33	0.64	0.46	0.000	0.33	0.64
Gauteng	0.24	0.000	0.17	0.34	0.24	0.000	0.17	0.34
Mpumalanga	0.41	0.000	0.29	0.57	0.41	0.000	0.29	0.57
Limpopo	0.31	0.000	0.22	0.43	0.31	0.000	0.22	0.43
**Geographical Type**								
Urban	**RC**				**RC**			
Rural	0.73	0.000	0.62	0.86	0.72	0.000	0.61	0.86
**Educational Attainment**								
No education	**RC**				**RC**			
Primary	1.86	0.000	1.46	2.38	1.88	0.000	1.47	2.40
Secondary	2.72	0.000	2.14	3.47	2.92	0.000	2.30	3.71
Higher	4.45	0.000	3.20	6.19	5.61	0.000	4.09	7.70
**Marital Status**								
Never Married	**RC**				**RC**			
Married	1.31	0.000	1.13	1.53	1.33	0.000	1.14	1.54
Divorced	2.53	0.000	1.60	4.02	2.58	0.000	1.63	4.09
Widowed	0.90	0.342	0.73	1.12	0.89	0.342	0.72	1.09
**Health insurance Cover**								
No	**RC**							
Yes	1.37	0.003	1.11	1.69				
**Wealth Index**								
Poor	**RC**				**RC**			
Middle	1.12	0.192	0.94	1.33	1.14	0.192	0.96	1.35
Rich	1.38	0.001	1.14	1.67	1.47	0.001	1.22	1.77
**Occupation**								
Not working	**RC**							
Professional/Formal	1.38	0.000	1.16	1.65				
Non-professional/Informal	1.24	0.016	1.04	1.48				
**Perceived health status**								
Poor	**RC**							
Average	0.80	0.025	0.66	0.97	0.82	0.025	0.68	0.99
Good	0.81	0.040	0.67	0.99	0.84	0.040	0.69	1.03
Excellent	0.67	0.003	0.51	0.88	0.71	0.003	0.55	0.92

Source: SADHS, 2016; RC – Reference category

Also, educational attainment has a significant association with Pap smear uptake, as the odds of being screened for cervical cancer among women having secondary and higher education were 2.7 and 4.4 times higher as compared to women who have no education (Table 3). Women who had health insurance cover had 13% higher odds of having Pap smear uptake compared to those who had no health insurance cover, and this was found to be statistically significant (UOR; 1.36, 95% CI; 1.11–1.68). For occupational status, women who are in the professional/formal sector had 14% higher odds of taking Pap smear test compared to those who are not working (AOR; 1.38, 95% CI; 1.14–1.69).

## Discussion

This study examined in an all-inclusive manner the background determinants of the use of cervical cancer screening among women of reproductive age in South Africa, with particular emphasis on individual proportion of geographical type demerit (urban and rural), occupation disadvantage (not working, professional and non-professional), wealth index drawback (poor, middle and rich), health insurance cover (yes and no), and educational attainment disadvantage (no education, secondary and higher). The study found that unmeasured irregularity in cervical cancer screening routine across age and population groups was significant through peripheral factors, as population characteristics are explained by significant differences. Such significant differences have been observed in several studies, where population characteristics have accounted for specific background unpredictability factors that determine cervical cancer screening utilization among women of reproductive age [6, 52, 53].

Generally, all the background factors for the study were found to be significantly associated with cervical cancer screening uptake at the individual level, a lattice of other factors (Model II). This observation is not unusual when all individual-level variables are considered simultaneously [8, 54, 55]. This is a key finding showing the significance of the multilevel models in examining classified structured datasets of the 2016 SADHS. Further, access to health facilities have been established to be an important determinant of cervical cancer screening among women [28, 32, 56], as population characteristics seem to be more significant in health matters. The identified significant individual-level factors were age group, population group, province, geographical type, educational attainment, marital status, health insurance, wealth index, occupation, and perceived health status. The consequences of these factors in cervical cancer screening uptake are well established in studies conducted in African countries [5, 57].

Our study contributes to the understanding of determinants and levels associated with cervical cancer screening uptake in South Africa, where the cervical cancer screening prevalence remains low [58, 59]. To the best of the authors’ knowledge, this is the first study that assesses population factors associated with cervical cancer screening uptake in South Africa using the 2016 SADHS. Our findings reveal that a significant number of women (64.46%) did not have an uptake of cancer screening; while the small fraction of the female population who had had a Pap smear were majorly found in Limpopo (25.96%) and KwaZulu-Natal (28.27%) provinces. Thus, lack of knowledge, younger age, lack of income, apprehension of Pap smear testing, and poor accessibility of health facilities’ screening services were significantly associated with low cervical cancer screening outcomes [4, 33, 60]. Consequently, in most industrialized countries, cervical cancer screening programmes have shifted from clinician-sampling to self-sampling for HPV testing, which has been proven to be equally accurate in combination with a follow-up Pap smear test. This implies that various health stakeholders can incorporate self-sampling HPV DNA testing into cervical cancer screening health educational programmes, especially at grassroots’ levels. Present studies have recommended self-sampling HPV DNA testing to be feasible, and may significantly improve cervical cancer screening uptake in South Africa [61–63].

Our study revealed that the prevalence of women who had Pap smear testing was higher among women with poor perceived health status, and also among those residing in Western Cape and North West provinces, respectively. A possible explanation for the provincial variation observed is that the urban settlements characteristically have higher socioeconomic status, less cultural conservatism, and easier access to health care services [64–66]. Corroborating the results of similar studies, our results demonstrate that women’s occupation and wealth index were positively associated with cervical cancer screening [5, 31, 57]. The lower outcome of cervical cancer screening among women of reproductive age may indicate a financial burden, which is a barrier to accessing cervical cancer screening services. Women with higher occupational status were more likely to undergo screening because these groups of women are most likely to own health insurance cover [1, 67]. Our study determined that individual-level factors such as women’s wealth index had a positive influence on cervical cancer screening behaviour and attitude, indicating that gender disparity, as assessed through wealth index, can affect screening services uptake.

Studies have indicated that women do not accumulate properties converted as wealth as much as men, resulting in a gender wealth gap [68, 69], as women are often perceived as passive and powerless; in addition, societies have apportioned wealth and assets ownership as mostly in the male domain. Besides, several myths and misinformation related to women that have undergone cervical cancer screening have been accepted generally [34], and consequently have various health implications among such women who will tend towards denial of life-threatening health problems [20, 21]. Empowering women with the right knowledge of the benefits of Pap smear testing is necessary and key to having prior information about health matters, especially with terminal diseases. Women who resided in urban settlements, comprising a higher proportion of women with secondary and higher educational attainment, were more likely to undergo screening. This finding is consistent with the findings of previous studies, suggesting that communities with a high concentration of educated women can increase the utilization of health care facilities, including cervical cancer screening services [70, 71]. Education is frequently associated with increased access to health care services and improved knowledge regarding health matters and behaviours. Increasing the proportion of educated women may facilitate the dissemination of knowledge and awareness to those with lower education, aiding them in accessing health services through informal social networks and contacts within their community space [18, 69].

Supporting our study objectives and previous findings of other studies [33, 59, 72], we found a positive association between the white female population group and cervical cancer screening. The possible explanation for this unexpected result is that the white population group may have higher expectations for health services and accessibility to health facilities. Other structural reasons may be gaining full access to transportation and finance to access cervical cancer screening services, which can influence screening behaviour among the white population group [33, 60]. Urban women were seen to have undertaken more Pap smear testing than rural women, as geographical type was significantly associated with Pap smear testing. It could be inferred that women in urban settlements may have access to sensitization programmes on Pap smear testing, health facilities and health insurance cover. Women in Western Cape Province were found to have undergone more Pap smear tests than women from Limpopo province. The outlying areas in South Africa play a role in sensitization of women on the benefits of cervical cancer, as these provinces are stratified as urban and rural settlements due to infrastructural facilities put in place in these areas.

Further research should include these potential factors associated with cervical cancer screening in the study design and analysis. As anticipated, health insurance coverage was strongly associated with cervical cancer screening outcomes, as our findings regarding the association between health insurance cover and Pap smear testing are consistent with the outcomes of previous studies [58, 59]. Our study findings prove that the adoption of a universal health insurance scheme ensuring equity in access to health care can largely enhance the possibility of cervical cancer screening use [6]. The cost of a Pap smear test may be a major impediment in cervical cancer screening uptake among women with meagre earnings, as this may further explain the low turn-out for cervical cancer screening among rural women [52, 59]. In a geographical area where poverty is high, alternative health services are given greater priority to out-of-pocket payments than preventive services [53, 59], and consequently, health insurance cover may potentially reduce the financial burden for rural women to have access to preventive health care services, including Pap smear testing.

In line with other studies, this study has shown a positive relationship between perceived health status and uptake of Pap smear testing. Women of reproductive age with consistent upkeep and maintenance may have regular dealings with health facilities and health care workers, including going for a Pap smear test when necessary [1, 59]. Specifically, in constrained settings, interaction with community health personnel after visitation to health facilities may increase women’s exposure to undertake preventive measures and encourage adoption of accurate health information on the benefits of cervical cancer screening [4]. Preceding studies have also validated that having a prescription from a doctor and treatment medic’s prescription and medicament was constantly found to be a strong predictor of adherence to cervical cancer screening [6, 53]. Our study has some prospective limitations. First, the cross-sectional study design restricted our capacity to draw underlying deductions for the relationship of individual-level factors with uptake of a Pap smear test, which require a longitudinal designs, but could not be determined. In addition, due to limited number of variables collected by the 2016 SADHS, we could not examine a full array of factors related to cervical cancer screening, particularly cultural factors including the quality of the health service and other factors related to user-friendliness of cancer screening facilities. The study also suggests the use of ethnographic methods that may unravel community factors that may influence the outcomes of cervical cancer screening.

## Conclusion and recommendation

Most women in South Africa demonstrated a low uptake of cervical cancer screening, as utilization of Pap smear testing among women of reproductive age in the study area was 35.5%. Despite that, when compared to previous studies conducted in countries within East Africa (Uganda – 20.6%; Kenya – 12.3%), West Africa (Nigeria – 13.5%; Benin – 0.6%) and Southern Africa (Namibia – 23.6%; Zimbabwe – 17.0%), there is still a high coverage of Pap smear testing among women of reproductive age in South Africa [73–77]. As implied in the literature, cervical cancer screening is associated with an increase in demographic factors that will influence the utilization of the services [33, 60]. Although in this study, demographic factors (such as age, educational attainment, health insurance cover, occupation, geographical type, and perceived health status) were significantly associated with utilization of cervical cancer screening services, yet in reality cervical cancer screening uptake is relatively low. Thus, these factors that result in cervical cancer screening services usage vary between individuals and from community to community [8, 54, 55]. The most basic conclusion is that there is a significant variation that exists in the population group and provinces in the use of cervical cancer screening services among women in South Africa.

The selected broad range of individual-level factors was not able to explain contextual variation as there is much more variation in cervical cancer screening among factors related to women’s utilization of cervical cancer screening services. Policies that look at women’s individual characteristics, such as promoting women employment, especially in professional occupations, higher educational level attainment and wealth accumulation, should continue to be implemented in order to increase Pap smear test uptake and achieve the universal health coverage goal. To mitigate those problems, there should be consistent monitoring of the cervical cancer detection and preventive actions among women through sensitization of health education, to modify women’s behaviours and attitude towards adopting cervical cancer screening. Health professionals and community health workers should promote educational and health recreational programmes provided by local councilors for individuals in their respective communities to have a prior knowledge of the dangers of not undergoing Pap smear testing.

Since early case detection through screening is the most cost effective activity for reducing the disease burden, reproductive health workers and policy stakeholders are needed to demonstrate more commitment in creating awareness about cervical cancer. Stakeholders in the Ministry of Health are needed to design and make Pap smear tests free for women through the establishment of more screening centres in different geographical areas of the provinces in South Africa. The existing Pap smear testing programmes mostly provided by non-governmental as well as faith-based organizations, which are majorly located in urban settlements, need to be decentralized and harmonized for greater efficiency in South Africa. Also, there is need to integrate the cervical cancer screening exercise into the mainstream health care services in the medical institutions. Women who are at least 15 years of age, particularly those with a family history of cervical cancer, must be encouraged to opt for cervical cancer screening at every available opportunity. Lastly, there is need to increase the number of medical professionals and community health workers with the requisite skills to conduct cervical cancer screening in South Africa.

### Implications for practice and/or policy

Our findings suggest that the implementation of health educational policies promoting cervical cancer screening through awareness and sensitization programmes can enhance the uptake of the testing by women. Thus, employment programmes should be targeted to provide occupation and job opportunities among women, as instituting income-generating programmes may intensify women’s intent in uptake of screening services. In addition, the impact of expanding improved health insurance coverage could be considerable. Health insurance coverage can possibly aid in the reduction of out-of-pocket health expenses for women of reproductive age, and empower them financially to claim and be able to access cervical cancer screening services. Also, health policymakers should address the problem of geographical disparities in the uptake of Pap smear testing; this can be achieved through effective approaches such as building up better cancer screening health facilities and health workers in rural settlements to minimize geographical inequity. Public health programmes must be intensified to target women of childbearing age about the advantages of early detection of cervical cancer and to encourage them to adopt preventive behavioural modifications. Furthermore, to increase the improvement of the overall coverage of Pap smear testing in order to attain optimum safeguarding of themselves against cervical cancer burden, self-testing and collecting samples of human papilloma virus can be a possible proposition for cervical cancer screening in future policy development.

## Data Availability

Data are from the Demographic and Health Survey and the dataset is open to qualified researchers free of charge. In order to access the data from DHS Measure, a written request was submitted to the DHS MACRO and permission was granted to use the data for this survey. To request access to the dataset, please apply at https://dhsprogram.com/data/dataset_admin/login_main.cfm?

## Abbreviations

CC: Cervical cancer
AOR: Adjusted odds ratio
CI: Confidence interval
HPV: Human papilloma virus
ORs: Odds ratio
SADHS: South Africa demographic health survey
Adjusted: A
Unadjusted: U
DNA: Deoxyribonucleic acid
STATA: Statistical analysis software
SSA: Sub-Saharan Africa
RC: Reference Category
